# Treatment of glomerular and tubular proteinuria in the nephrotic range in a female cat: case report

**DOI:** 10.29374/2527-2179.bjvm012825

**Published:** 2026-03-19

**Authors:** Maria Vitória dos Santos Pascoal, Guilherme Sena, Stephanie Favato de Azevedo, Juliana de Moraes Intrieri, Heloísa Justen Moreira de Souza

**Affiliations:** 1 Instituto de Veterinária, Universidade Federal Rural do Rio de Janeiro, Seropédica, RJ, Brazil.; 2 Universidade Federal Fluminense, Niterói, RJ, Brazil.; 3 Programa de Pós-Graduação em Ciências Veterinárias, Universidade Federal Rural do Rio de Janeiro, campus Seropédica, RJ, Brazil.; 4 Departamento de Medicina e Cirurgia Veterinária, Universidade Federal Rural do Rio de Janeiro, Seropédica, RJ, Brazil.

**Keywords:** proteinuria, glomerulopathy, anasarca, proteinúria, glomerulopatia, anasarca

## Abstract

The objective is to serve as a descriptive and educational report regarding the case of an eight-year-old spayed mixed-breed cat with nephrotic-range tubular and glomerular lesion, with no history of prior medication use or comorbidities, the main complaint being prostration and sudden weight gain. Physical examination revealed anasarca, moderate dyspnea and paradoxical breathing. Imaging tests revealed bicavitary effusion and subcutaneous edema. Laboratory analyses revealed serum creatinine equal to 3.0 mg/dL, severe hypoalbuminemia of 0.8g/dL, urine specific gravity equal to 1.020 and intense proteinuria (UPC ratio= 13.09). Urinary electrophoresis confirmed proteinuria with both glomerular and tubular involvement. This led to the suspicion of nephrotic syndrome with mixed-origin lesions. Treatment included furosemide, dexamethasone, benazepril, omega-3 fatty acid, mirtazapine and renal diet, in addition to procedures such as paracentesis for drainage. Progressive clinical improvement was observed, with resolution of anasarca by day 21, normalization of albumin by day 37 and a reduction of up to 93.85% in proteinuria nine months after the end of treatment. The patient remained stable, with no recurrence of symptoms. The therapeutic conclusions are limited by the single-case design, although continuous clinical and laboratory monitoring was implemented, aiming to adjust therapeutic management.

## Resumo

Objetiva-se relatar, de forma descritiva e educacional, o caso de uma gata, sem raça definida, castrada, com oito anos, com lesão em faixa nefrótica tubular e glomerular, sem uso de medicações ou comorbidades pregressas, com queixa de prostração e aumento súbito de peso. Ao exame físico, observaram-se anasarca, dispneia moderada e respiração paradoxal. Os exames de imagem revelaram efusão bicavitária e edema subcutâneo. As análises laboratoriais evidenciaram creatinina sérica igual a 3.0 mg/dL, hipoalbuminemia severa (0,8g/dL), densidade urinária igual a 1020 e proteinúria intensa (RPCU = 13,09). A eletroforese urinária confirmou proteinúria com comprometimento glomerular e tubular. O que levou a suspeita de síndrome nefrótica, com lesão de origem mista. O tratamento incluiu furosemida, dexametasona, benazepril, ácidos graxos ômega-3, mirtazapina e dieta renal, além de procedimentos como paracentese para drenagem. Observou-se melhora clínica progressiva, com resolução da anasarca até o 21º dia, normalização da albumina no 37º dia e redução de até 93,85% da proteinúria aos nove meses após o término do tratamento. A paciente manteve-se sem recidiva dos sintomas. As conclusões terapêuticas são limitadas pelo fato de se tratar de um caso único, ainda que se tenha implementado monitoração clínica e laboratorial contínua, com revisões mensais estruturadas, visando ajustar o manejo terapêutico.

## Introduction

Glomerulopathies are uncommon diseases in felines. The clinical course is silent and can evolve either slowly or acutely to the deterioration of renal function (Ilgün et al., 2024). Among the causes, the use of non-steroidal anti-inflammatory drugs (NSAIDs), such as ibuprofen, and the formation of immune complexes resulting from infectious diseases are prominent in cats (Herrero et al., 2022; Rossi et al., 2019).

The antigen-antibody complexes are deposited on the glomerular basement membrane, which activates the complement system and triggers damage to glomerular cells, resulting in nephrotic syndrome (Rossi et al., 2019).

Nephrotic syndrome (NS) is characterized by a set of laboratory changes, such as hypoalbuminemia, hypercholesterolemia, marked and persistent proteinuria (UPC ratio > 3.5), which may or may not be accompanied by azotemia. Clinical findings include inappetence, weight gain, cavitary effusions, and/or peripheral edema. This condition can rapidly evolve into end-stage renal failure or lead to the patient's death due to complications (Kamiie et al., 2011).

The presumptive diagnosis is established by evaluating biomarkers of glomerular injury, such as urinary electrophoresis, which allows distinguishing whether protein loss results from glomerular lesions, tubular lesions, or both, based on the molecular weight and characteristics of the excreted proteins (Ferlizza et al., 2017). However, the definitive diagnosis requires histopathological analysis of a renal biopsy (Broadbridge et al., 2022). Thus, this article reports the clinical case of a cat with nephrotic syndrome, presenting glomerular and tubular proteinuria in the nephrotic range.

## Case report

A spayed, 8-year-old domestic short-haired cat (DSH) weighing 4.55 Kg, was referred for consultation with a complaint of sudden weight gain, prostration, inappetence, and peripheral edema. The echocardiographic examination revealed a mild interventricular septal hypertrophy with preserved cardiac chamber dimensions.

Upon our evaluation (Day 0), the feline patient suffered from severe and generalized fluid accumulation in the subcutaneous tissue, in addition to moderate expiratory dyspnea with paradoxical breathing. On initial assessment, the respiratory rate was 68 breaths per minute and the heart rate was 180 beats per minute. Auscultation revealed muffling of heart sounds and respiratory sounds. Indirect systolic blood pressure ranged from 120 to 130 mmHg. On palpation, positive pitting edema (Godet sign) was present on the face, cervical region, thorax, abdomen, and limbs. The rectal temperature was 39.5°c.

**Figure 1:**
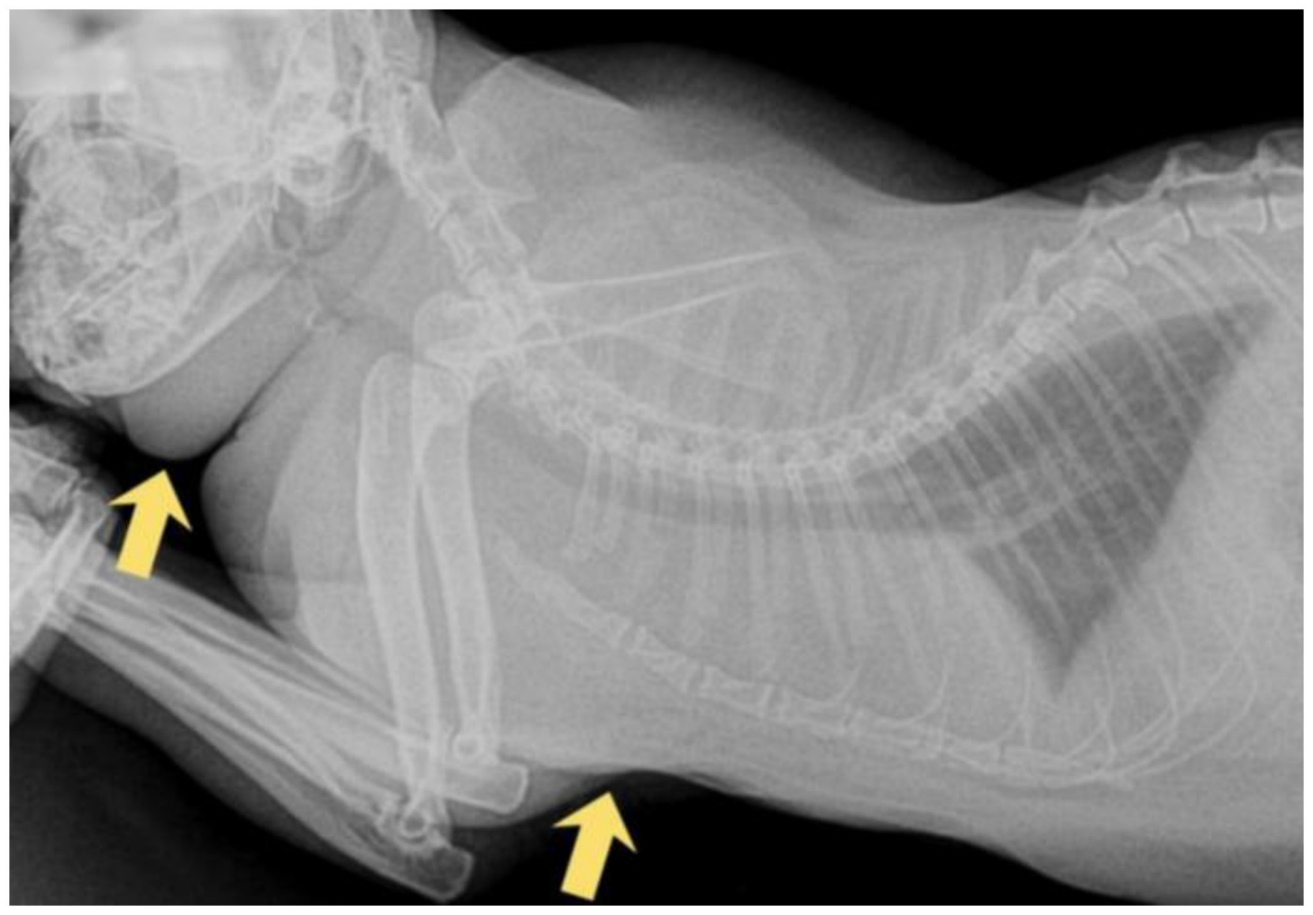
Lateral thoracic radiograph of an 8-year-old cat with suspected nephrotic syndrome. Note the pleural effusion and fluid accumulation in the subcutaneous tissues of the cervical and ventral thoracic regions (yellow arrows). Source: Personal archive.

The thoracic radiographic evaluation revealed moderate effusion in both hemithoraces, in addition to fluid accumulation in the subcutaneous tissue and the ventral cervical region (Figure 1).

The abdominal ultrasonography findings were consistent with a large amount of free fluid, chronic bilateral nephropathy, showing discrete changes in contour, cortico-medullary definition, and echotexture (left kidney: 3.08 cm, with a cortical cyst measuring 0.37 x 0.38 cm; right kidney: 3.35 x 2.29 cm).

**Figure 2:**
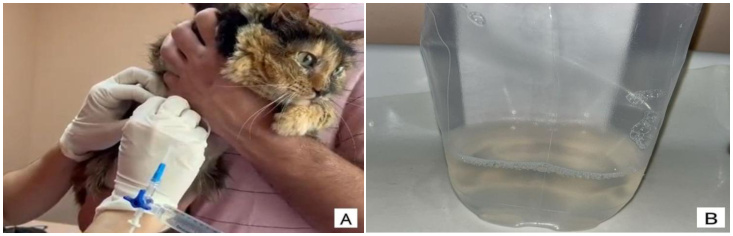
Patient with suspected nephrotic syndrome undergoing thoracocentesis. A – Gentle restraint by the owner in an upright position, where fluid was aspirated from the lower portion of the thorax at the level of the seventh ventral intercostal space using a 23G scalpel, a three-way stopcock, and a 10 mL syringe, in a closed circuit. B – Observation of 150 mL of translucent, acellular fluid, characterized as a simple transudate. Source: Personal archive

The patient underwent bilateral thoracocentesis (Figure 2A), and a total volume of 150 mL of translucent, colorless fluid, characterized as an acellular simple transudate, was drained (Figure 2B). A total of 110 mL was drained from the right side and 40 mL from the left side. Furthermore, during manipulation, the patient also transuded fluid from its subcutaneous tissue.

Laboratory analysis of the blood samples demonstrated serum hypoproteinemia of 3.3 g/dL (ref: 5.4-7.8 g/dL), due to severe hypoalbuminemia of 0.8 g/dL (ref: 2.1-3 g/dL). Increases were observed in serum creatinine values, equal to 3.0 mg/dL (ref: 0.5-1.9 mg/dL), urea of 97 mg/dL (ref: 30-60 mg/dL), and phosphorus of 3.7 mg/dL (ref: 2.5-6.1 mg/dL). The remaining serum biochemistry parameters remained within the normal range, with triglycerides of 34 mg/dL (ref: 10-114 mg/dL), cholesterol of 185 mg/dL (ref: 80-250 mg/dL), potassium of 4.6 mmol/L (ref: 3.8-5.5 mmol/L), and sodium of 156 mmol/L (ref: 147-156 mmol/L).

The urinalysis revealed a density of 1.035 (ref: 1.045–1.060), with no sediment or hematuria, a pH of 5 (ref: 5.5–7.0), and significant proteinuria. Evaluation of the urine protein-to-creatinine ratio (UPC) showed a remarkably elevated rate of 13.09 (ref: > 0.4)

In the analysis performed using the polymerase chain reaction (PCR) technique, the presence of 11 antigens associated with the formation of immune complexes was not detected. These antigens included: *Mycoplasma haemofelis, Anaplasma* spp., *Babesia* spp., Feline Coronavirus (FCoV*), Bartonella* spp., *Cytauxzoon* spp., *Ehrlichia* spp., feline immunodeficiency virus (FIV), feline leukemia virus (FeLV), *Candidatus Mycoplasma haemominutum*, and *Candidatus Mycoplasma turicensis.*

Nonspecific treatment was initiated with furosemide at a dose of 2 mg/kg every 24 hours for 10 days, subsequently reduced to 1 mg/kg for an additional 10 days. This was associated with massotherapy sessions for drainage of subcutaneous fluid. The patient showed improvement in the respiratory condition.

Activation of the Renin-Angiotensin System is a factor that potentiates renal protein loss, so an angiotensin-converting enzyme (ACE) inhibitor was employed. Benazepril was instituted at a dose of 0.25 mg/kg every 24 hours throughout the 9 months of treatment. A therapeutic renal diet, which provides reduced levels of phosphorus, an adjusted amount of sodium, and high-digestibility proteins with high biological value was also recommended.

The administration of omega-3 at a dose of 1.1 IU/g of fish oil every 24 hours for 21 days was recommended to promote reduction of glomerular capillary pressure and decrease the presence of free radicals. Initially, dexamethasone at an immunosuppressive dose of 0.2 mg/kg was administered in a single dose. Due to the complaint of inappetence, mirtazapine was instituted at 2 mg/cat every 48 hours for 15 days.

Serial clinical and laboratory follow-up of the patient was conducted for 9 months. After 7 days of treatment, the animal lost 360g of body weight, showing only mild limb edema. However, an additional 410 mL of translucent fluid was still drained via paracentesis (200 mL from the right hemithorax and 210 mL from the abdomen).

The arterial blood pressure remained around 130 to 140 mmHg throughout the monitoring period. There was an improvement in total protein, with value equal to 3.8 g/L (ref: 5.4–7.8 g/dL), and albumin at 1 g/dL (ref: 2.1–2 g/dL). The urine protein-to-creatinine ratio (UPC) was 11.013, which remained severely elevated.

**Figure 3:**
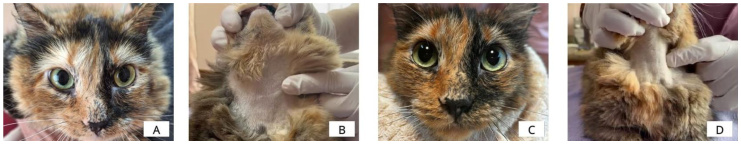
A patient with 8 years old, DSH, with suspected nephrotic syndrome. A – Note facial swelling at the first consultation (Day 0). B – Appearance of the ventral cervical region; during inspection with the index finger, significant subcutaneous edema and a positive pitting sign were noted. C and D – Note the reduction in swelling of the face and ventral cervical region 21 days after treatment initiation. Source: Personal Archive.

After 21 days of treatment, the patient had total weight loss of 1.130 kg, with no peripheral edema present. The patient was much more active at home, with improved appetite and respiratory pattern (Figure 3 A-D).

The total protein increased to 4 g/dL (ref: 5.4–7.8 g/dL), and albumin increased to 1.5 g/dL (ref: 2.3–3 g/dL). Proteinuria remained severe, with a urine protein-to-creatinine ratio (UPC) value of 9.498 (ref: > 0.4).

**Figure 4:**
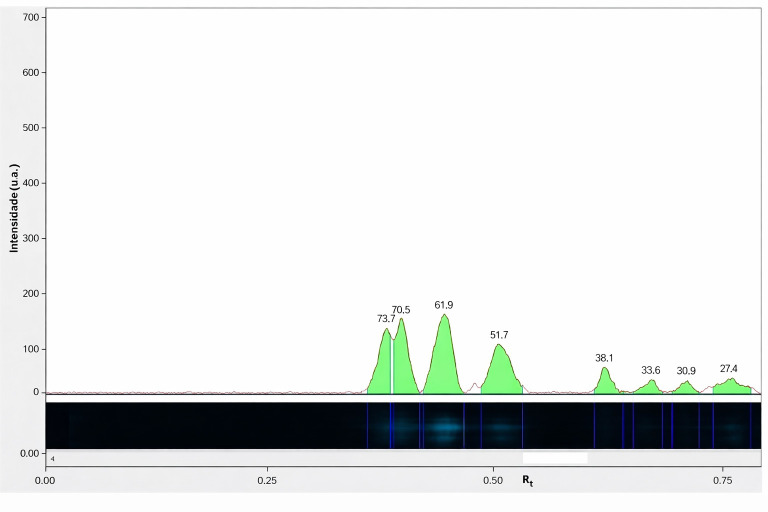
Graph representing urine protein electrophoresis based on its molecular weight. It is possible to observe 8 bands of both high molecular weight and low molecular weight, characterizing the lesion as being of glomerular and tubular origin. Source: Personal Archive.

A urine sample was sent for urine protein electrophoresis (Day 37), where it was possible to identify 8 bands, representing both high molecular weight proteins (more than 60 kDa), which corresponded to 61.6% of the total, and low molecular weight proteins (less than 60 kDa), which totaled 38.4% (Figure 4). The electrophoretic profile confirmed the impairment of the glomerular barrier and the renal tubules, which demonstrated the mixed origin of the nephrotic syndrome.

**Figure 5:**
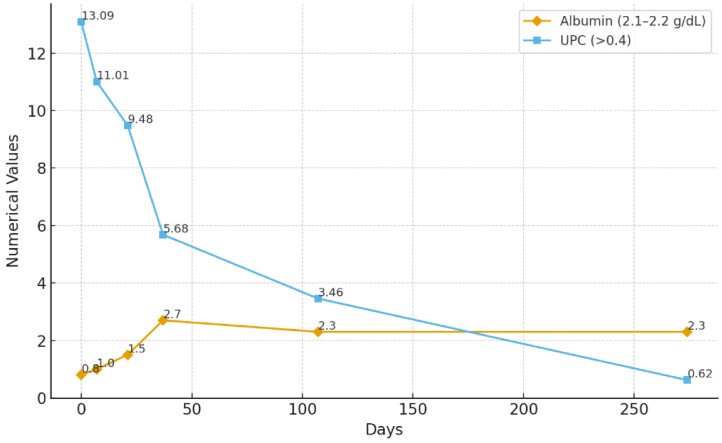
Graph representation of the serial monitoring of serum albumin levels (orange diamonds) and urine protein/creatinine ratio (blue squares), during 9 months of follow-up. Observe the elevation of albumin due to lower urinary loss. Likewise, note the drop in urinary protein values. Source: Personal archive

The patient did not present clinical alterations, such as edema, effusion, loss of appetite, or prostration for 9 months after the first consultation. During this period, creatinine remained between 2.5 and 3.3 (ref. 0.5-1.9 mg/dL), and urea between 84 and 118 mg/dL (ref. 30-60 mg/dL), which classifies it as having stage III renal disease. Serum total protein was equal to 6.2 g/dL (ref. 5.4-7.8 g/dL) and albumin was 2.3 g/dL (ref. 2.1-3 g/dL). Urinary density remained stable at 1.020 (ref. 1015-1045). The UPC ratio dramatically declined to 0.82 (ref. >0.4), a 93.85% decrease compared to the initial value (Figure 5). In serial urine cultures, there was no growth of microorganisms. The complete blood count tests remained stable throughout the treatment, with no need for interventions.

## Discussion

Glomerular and tubular lesions are frequently associated with infectious processes with immune complex neoplastic, toxic, or cardiopathic formation (Herrero et al., 2022). In the present case, these possible causes were not identified. The patient was not receiving prior medication, and had no contact with substances that could be toxic. An extensive evaluation through the polymerase chain reaction technique for the identification of 11 infectious agents that could lead to immune complex formation in cats did not produce comprehensive results. The echocardiographic examination revealed only mild septal hypertrophy, without functional repercussion. Due to the non-detection of possible causes, the suspected diagnosis was idiopathic nephrotic syndrome.

Even in cases of undefined etiology, structural or inflammatory changes in any component of the glomerular filtration barrier can compromise its hydrostatic and electrostatic properties, allowing the abnormal passage of plasma proteins — especially albumin — through the urine (Ren et al*.*, 2020; Rossi et al., 2019). Imaging exams and the values of serum urea and creatinine concentrations, even after partial control of proteinuria, indicate chronic renal injury compatible with stage III renal disease (Rayhel et al., 2020).

Since the patient did not have previous records, it was difficult to determine whether the renal injury occurred secondarily to the increase in UPC ratio or if they already had renal function compatible with stage III before the event. Serial monitoring of the urine protein/creatinine ratio and urine protein electrophoresis are valuable tools for assessing therapeutic response and the origin of proteinuria (Wannenburg, 2024). The electrophoretic profile in this case indicated protein loss of mixed origin — glomerular and tubular — and the severely elevated UPC ratio was consistent with the suspicion of nephrotic syndrome (Herrero et al., 2022).

The adopted therapy had as its main objective reducing the proteinuria and clinical symptomatology. The administration of furosemide, although contraindicated in patients with renal impairment, was necessary due to the severity of the edema and bicavitary effusion, which compromised ventilation and represented an imminent risk of death (Shafiee et al., 2021). The treatment was carried out under strict monitoring of renal and hydroelectrolytic parameters, which allowed the control of fluid overload.

The use of immunosuppressive therapy, such as dexamethasone, is generally recommended for management of glomerulonephritis due to the reduced inflammatory response and the partial restoration of the integrity of the glomerular barrier (Kamiie et al., 2011), although there is one report indicating that spontaneous remission can occur without the use of this medication class in felines (Broadbridge et al., 2022).

It is known that multifactorial hypercoagulability can occur in patients with glomerular diseases, with the main factors being related to the renal loss of antithrombin III, hyperfibrinogenemia, and platelet hypersensitivity (Brown et al., 2013). We chose not to institute antiplatelet therapy, since the patient was already too anorexic for the use of clopidogrel, in addition to not demonstrating elevations in blood pressure levels or platelet alterations in recurrent analyses. However, in patients with conditions favorable to thromboembolism (high UPC ratio, hypertension, hypoalbuminemia), the use of anticoagulant therapy and thromboprophylactics is indicated (Brown et al., 2013).

It is suggested that the use of an angiotensin-converting enzyme (ACE) inhibitor contributed to the reduction of proteinuria by decreasing intraglomerular pressure and protein filtration, thereby reducing UPC levels (Rayhel et al., 2020). The adoption of a diet with phosphorus restriction, moderate amounts of sodium, and selected proteins is recommended to attenuate renal injury, as described by Ilgün et al. (2024).

## Conclusion

This report illustrates a clinical and laboratory stabilization in the cat, with sudden weight gain, generalized edema, hypoalbuminemia, and severe proteinuria. The exponential increase in serum albumin and a 93.85% reduction in the urine protein/creatinine ratio at the end of 9 months suggests that the treatment employed based on ACE inhibitors, dietary modification, fatty acid supplementation, diuretics, and corticosteroids may have been the reason for the effectiveness in the multifactorial management of this nephrotic-range tubular and glomerular lesion, compatible with the suspicion of idiopathic nephrotic syndrome.
